# Determinants Associated with COVID-19 Vaccination among Korean Adults: Based on Andersen’s Model

**DOI:** 10.3390/bs14100905

**Published:** 2024-10-08

**Authors:** Eungyeong Kim

**Affiliations:** Department of Nursing, Kunsan National University, Gunsan 54150, Republic of Korea; egkim@kunsan.ac.kr; Tel.: +82-63-469-1994

**Keywords:** COVID-19, adolescent, vaccination, Andersen model

## Abstract

COVID-19 vaccination is a critical public health measure to control the pandemic, but disparities in vaccination uptake remain a concern. This study investigates the determinants of COVID-19 vaccination among Korean adults using the Andersen model. Data from 231,784 participants in the community health survey were analyzed using chi-square testing and logistic regression. The risk of non-vaccination was higher among those aged 19–64 (95% CI: 1.52–1.74), males (95% CI: 1.11–1.24), the unemployed (95% CI: 2.21–2.47), unmarried individuals (95% CI: 1.12–1.24), those with unmet healthcare needs (95% CI: 1.41–1.67), recipients of national basic livelihood guarantees (95% CI: 1.45–1.73), those with lower subjective health (95% CI: 1.20–1.30), individuals with depression (95% CI: 1.28–1.44), current smokers (95% CI: 1.13–1.30), and those skipping breakfast (95% CI: 1.04–1.16). Conversely, the risk was lower for those with less than a high school education (95% CI: 0.72–0.81), individuals with psychological concerns about infection (0.87, 95% CI: 0.82–0.92) or public criticism (0.91, 95% CI: 0.86–0.97), individuals with chronic diseases (95% CI: 0.64–0.72), and current alcohol consumers (95% CI: 0.52–0.58). These findings underscore the need for targeted intervention strategies and support systems to promote vaccination in vulnerable populations. Further research should explore the long-term impact of these interventions on vaccination uptake.

## 1. Introduction

The onset of COVID-19 in January 2020 heralded an unparalleled global health crisis, leading to widespread fatalities and significant lifestyle changes. The virus’s highly contagious nature, coupled with the emergence of mutated strains, continues to pose concerns about sustained transmission and enduring societal impacts [1]. Despite global vaccination efforts, COVID-19 remains a substantial challenge. Vaccination is pivotal not only for preventing infection but also for reducing the severity and risk of death from the virus [2]. While surpassing the World Health Organization’s vaccination target, there is an ongoing need to investigate factors influencing vaccination rates, particularly among the unvaccinated population [3]. As new infectious diseases persist, research becomes crucial to enhance community vaccination rates.

Prior studies have applied Andersen’s Model to discern factors affecting vaccination intentions [4]. The Andersen model, designed to predict individual use of medical services, categorizes factors into predisposing, enabling, and need factors [5]. Predisposing factors encompass demographic and sociological characteristics, enabling factors to cover aspects influencing the ability to use medical services and need factors pertaining to physical and mental health challenges [5].

In a study examining college students’ COVID-19 vaccination intentions applying the Andersen model, factors such as demographic characteristics, the influence of peers, psychological predispositions, the acquisition of COVID-19 vaccine information, vaccine literacy, health status, and COVID-19-related experiences were considered [3]. Psychological predisposition significantly influenced college students’ COVID-19 vaccination intentions [3]. International studies have indicated lower vaccination intentions among women, younger individuals, those with lower education, and individuals in non-medical majors and occupations [6,7]. A prior investigation into factors influencing COVID-19 vaccination status among the elderly in China revealed that women, unmarried individuals, urban residents, and those with chronic diseases are less likely to receive the COVID-19 vaccine [8].

Despite numerous studies on COVID-19 in Korea focusing on epidemiology, vaccine effectiveness, and vaccination intentions for specific groups, there is a scarcity of research identifying factors related to the actual vaccination of the entire Korean population. This study aims to fill this gap by applying the Andersen model to national-scale survey data, seeking to uncover factors influencing COVID-19 vaccination and contribute to the development of community programs aimed at improving vaccination rates. Additionally, it is important to address factors such as concerns about side effects, confidence in vaccine effectiveness, influence from peers and family, and policies regarding free vaccine provision to ensure a comprehensive understanding of vaccination behaviors and hesitancy.

### Anderson Model

The Andersen model, widely used in healthcare access studies, was selected for this research because it offers a comprehensive framework for understanding the multi-dimensional factors that influence health behaviors, such as COVID-19 vaccination. The model divides these factors into three categories: predisposing factors (e.g., demographic and psychological characteristics), enabling factors (e.g., resources or barriers to care), and need factors (e.g., health status and perceived need for care). These categories align well with the determinants explored in this study. Previous research has demonstrated the Andersen model’s effectiveness in examining vaccination behavior, particularly in understanding how individual characteristics, access to healthcare, and perceived needs influence vaccination decisions. By utilizing this model, we aim to identify the various factors that contribute to COVID-19 vaccination uptake among Korean adults.

## 2. Materials and Methods

### 2.1. Data and Sample

This study utilized primary data from the 2022 Korea Community Health Survey (KCHS) conducted by the Korea Disease Control and Prevention Agency between 16 August and 31 October 2022. The KCHS is an annual cross-sectional survey that employs a multistage, stratified area probability sampling method to select participants from civilian, non-institutionalized Korean households. Sampling is based on geographic area, age, and sex, ensuring representative data across different demographic groups. Data collection was performed through one-on-one in-person interviews using standardized questionnaires administered by trained surveyors.

For the purposes of this study, participants who reported receiving a COVID-19 vaccination were initially considered. However, individuals who reported being unvaccinated were excluded from the final analysis. After applying these exclusion criteria, a total of 231,784 individuals were retained in the final dataset.

### 2.2. Measurement

#### 2.2.1. Predisposing Factor

Predisposing factors refer to the demographic and psychological characteristics of individuals that may influence health behavior. In this study, age was categorized into two groups: 19–64 and 65 or older. Gender was classified as either male or female. Job status was divided into those with an occupation and those without. Spouse status was categorized based on whether participants had a spouse or not, including those who were never married, divorced, or widowed. Education level was classified as either below high school graduate or college and above. Lastly, psychological concerns related to COVID-19 were measured by asking participants to rate their level of concern in three specific areas: (1) concern about infection, (2) concern about public criticism, and (3) concern about economic damage.

In this study, psychological concerns related to COVID-19, such as fear of infection and economic anxiety, were considered part of predisposing factors. This is in line with the Andersen model, which allows for psychological characteristics to be included as individual-level factors influencing health behavior. Although these factors could also be viewed sociologically, they are incorporated here as predisposing influences based on individual perceptions and experiences.

#### 2.2.2. Enabling Factors

Enabling factors refer to conditions that either facilitate or hinder access to healthcare services. In this study, unmet healthcare needs were assessed by asking participants, “Over the past year, have you ever felt that you could not or did not access medical services when needed?” Responses were categorized as either “Yes” or “No”. The status of being a recipient of the national basic livelihood guarantee, a form of government-provided social assistance, was also considered. Additionally, region of residence was categorized based on participants’ living locations, grouped as either urban or rural areas.

#### 2.2.3. Need Factors

Need factors most directly reflect access to medical care by highlighting disease characteristics. In this study, subjective health, stress, depression, chronic diseases, current smoking, current alcohol drinking, and eating breakfast were included. Subjective health was categorized as “very bad, bad, normal, good, and very good” and later reclassified as “above good” and “below normal”. Depression was assessed using the Patient Health Questionnaire-9 (PHQ-9), recommended with a cutoff score of 5 [9] (Park et al., 2010). Chronic diseases included diagnosed hypertension and diabetes. Current smoking was determined by the question: “Do you smoke now?” Current alcohol drinking refers to those who consumed alcohol within the past month.

### 2.3. Statistical Analysis

Statistical analyses were performed using IBM SPSS Statistics v21.0 (IBM Corp., Armonk, NY, USA). Categorical variables were presented as proportions and compared using chi-square tests to identify differences between groups. Binary logistic regression was used as the outcome variable (vaccination status), which was dichotomous.

Both univariate and multivariate logistic regression analyses were conducted to examine the factors associated with COVID-19 vaccination. Univariate logistic regression was first employed to assess the crude relationships between each independent variable and the dependent variable (COVID-19 vaccination status). This step allowed us to identify the raw associations without adjusting for potential confounders.

Following the univariate analysis, multiple logistic regression was performed to adjust for confounding variables and determine the independent effects of each predictor on vaccination status. Variables that were significant in the univariate analysis (*p* < 0.05) were included in the multivariate model. The results were presented as odds ratios with 95% confidence intervals.

## 3. Results

The outcomes presented in Table 1 illustrate an analysis of COVID-19 vaccination patterns concerning predisposing, enabling, and need factors. Among the participants, 3.4% reported being unvaccinated. The frequencies and percentage distributions of each categorical variable related to COVID-19 vaccination were derived using the χ^2^ test. Non-vaccination rates were notably elevated among individuals aged 19–64 (71.4%), females (56.1%), those without an occupation (55.5%), individuals without a spouse (42.9%), those with education below the high school level (55.4%), individuals with psychological concerns about COVID-19 infection (60.3%) and economic repercussions (50.6%), those lacking psychological concerns about COVID-19 public criticism (55.5%), individuals with experienced unmet healthcare needs (9.6%), recipients of national basic livelihood guarantees (9.5%), urban residents (60.9%), individuals reporting higher subjective health (35.2%), those experiencing stress (74.8%), individuals with depression (24.8%), those without chronic diseases (73.3%), individuals who currently smoke (18.2%), those not currently drinking alcohol (73.3%), and those not having breakfast (60.4%).

Logistic regression analyses were employed to assess the impact of predisposing, enabling, and need factors on COVID-19 vaccination (Table 2). Both univariate and multivariate models were used to compare the crude and adjusted effects of each predictor. The multivariate logistic regression model achieved statistical significance (χ^2^ = 2968.80, df = 18, *p* < 0.001), indicating that the collective independent variables reliably predicted the likelihood of COVID-19 vaccination.

In the univariate logistic regression analysis, the odds of COVID-19 non-vaccination were 1.31 times higher for individuals aged 19–64 (95% CI: 1.25–1.38) compared to those aged 65 and older (reference group). Males had 1.08 times higher odds of non-vaccination (95% CI: 1.03–1.13) compared to females (reference group). Individuals without an occupation had 0.46 times lower odds of non-vaccination (95% CI: 0.44–0.48), with those in an occupation serving as the reference group. The odds of non-vaccination were also 1.47 times higher for individuals without a spouse (95% CI: 1.40–1.54) compared to those with a spouse (reference group). Moreover, individuals with a high school education or lower had 1.28 times higher odds of non-vaccination (95% CI: 1.22–1.34) compared to those with a college degree or higher (reference group). Furthermore, psychological concerns, such as fear of COVID-19 infection, were associated with a 1.21 times increase in the odds of non-vaccination (95% CI: 1.15–1.26) compared to those without such concerns.

In the multivariate logistic regression analysis, after adjusting for all variables, the risk of COVID-19 non-vaccination increased by 1.63 times for individuals aged 19–64 (95% CI: 1.52–1.74) compared to those aged 65 or older (reference group). Males had 1.17 times higher odds of non-vaccination (95% CI: 1.11–1.24) compared to females (reference group). Individuals not in occupation had 2.34 times higher odds of non-vaccination (95% CI: 2.21–2.47) compared to those with an occupation (reference group). The risk of non-vaccination was also 1.18 times higher for individuals without a spouse (95% CI: 1.12–1.24) compared to those with a spouse (reference group). In contrast, individuals with a high school education or lower had 24% lower odds of non-vaccination (OR = 0.76, 95% CI: 0.72–0.81) compared to those with a college degree or higher (reference group). Notably, psychological concerns, such as fear of COVID-19 infection, decreased the odds of non-vaccination by 13% (OR = 0.87, 95% CI: 0.82–0.92) compared to individuals without such concerns, while public criticism similarly reduced the odds by 9% (OR = 0.91, 95% CI: 0.86–0.97).

In terms of enabling factors, individuals with unmet healthcare needs had 1.54 times higher odds of non-vaccination (95% CI: 1.41–1.67) compared to those without unmet healthcare needs (reference group). Similarly, recipients of national basic livelihood guarantees had 1.59 times higher odds of non-vaccination (95% CI: 1.45–1.73) compared to non-recipients (reference group).

Regarding need factors, the risk of non-vaccination was 1.27 times higher for individuals with lower subjective health (95% CI: 1.20–1.30) compared to those reporting good health (reference group). Individuals with depression had 1.36 times higher odds of non-vaccination (95% CI: 1.28–1.44) compared to those without depression (reference group). Current smokers had 1.21 times higher odds of non-vaccination (95% CI: 1.13–1.30) compared to non-smokers (reference group), while not eating breakfast increased the odds of non-vaccination by 1.10 times (95% CI: 1.04–1.16) compared to those who ate breakfast regularly (reference group). Conversely, individuals with chronic diseases had 32% lower odds of non-vaccination (OR = 0.68, 95% CI: 0.64–0.72) compared to those without chronic diseases (reference group), and those who consumed alcohol had 45% lower odds of non-vaccination (OR = 0.55, 95% CI: 0.52–0.58) compared to non-drinkers (reference group).

## 4. Discussion

This study examines the determinants of COVID-19 vaccination in South Korea using data from the 2022 Korea Community Health Survey (KCHS). Given that vaccination is one of the most cost-effective measures during public health crises, the Andersen model was employed to provide a comprehensive framework for assessing the factors influencing vaccination decisions. The findings are consistent with previous studies while also revealing new insights into vaccination behavior in South Korea.

Predisposing factors, such as age, gender, job status, marital status, education level, and psychological concerns about COVID-19, significantly impact COVID-19 vaccination. Individuals over 65, females, those employed, and those with spouses exhibited a lower risk of being unvaccinated, aligning with previous findings [10,11]. This trend could be attributed to the national vaccination program’s focus on individuals aged 65 and older, which successfully increased vaccination rates in this group. Interestingly, individuals with higher education (college level and above) showed a greater risk of being unvaccinated. This result contradicts some prior findings [11], suggesting that more research is needed to explore the influence of education on vaccine hesitancy. Psychological concerns related to COVID-19 underscore the need for psychological support programs, considering the psychological impact on the vaccination group. In preparing COVID-19 countermeasures, addressing psychological and social aspects, tailored to the varying levels of psychological concern, is imperative. Effective intervention and support systems are vital as community residents’ psychological concerns can influence their health and behavior [12]. Tailored psychological support and information campaigns should address these concerns to improve vaccination rates [13].

Enabling factors like unmet healthcare needs and recipients of national basic livelihood guarantees were associated with higher risks of being unvaccinated. These findings underscore the necessity of targeted interventions for socially vulnerable populations, such as economically disadvantaged groups, who may face barriers to accessing healthcare [11]. Providing reliable information on the safety and efficacy of vaccines, coupled with support systems, could improve vaccination coverage in these communities. Social distancing measures and the pandemic’s broader social impacts have further exacerbated the challenges for these populations [14].

Need factors, including subjective health, depression, current smoking, and skipping breakfast, were also associated with a higher risk of being unvaccinated. People reporting poor subjective health or depression often display vaccine hesitancy [15], necessitating motivational and educational programs [16]. The association between smoking and vaccine distrust reflects the need for targeted interventions aimed at smokers [17], as smoking may contribute to vaccine hesitancy [18]. Skipping breakfast, often linked to poor nutrition, could impair immune function [19] and thus influence attitudes toward vaccination [20]. Interestingly, those with chronic diseases were less likely to be unvaccinated, suggesting that they understand the preventive benefits of vaccination, which is consistent with previous studies [21]. However, the lower risk of being unvaccinated among current drinkers contradicted earlier research [22], highlighting the need for further investigation.

### 4.1. Strengths and Limitations

This study benefits from the use of a large, nationally representative dataset, enhancing the generalizability of its findings. The application of the Andersen model also allows for a structured analysis of factors influencing vaccination behavior. However, a limitation is the absence of cognitive factors, such as knowledge and attitudes, which could further explain vaccine hesitancy. Additionally, as the COVID-19 pandemic evolves and more information becomes available, public perceptions of vaccines may change, which this study could not account for. Future research should include longitudinal data to capture these shifts.

### 4.2. Implications for Policy and Practice

The findings from this study highlight several areas for future action. First, psychological concerns and barriers to healthcare access must be addressed through targeted intervention strategies, particularly for economically vulnerable populations. Educational campaigns should focus on debunking myths about vaccines and promoting the benefits of vaccination, particularly among those with higher education and those who engage in unhealthy behaviors like smoking. Public health strategies should also integrate tailored support for individuals with poor mental health and unmet healthcare needs, which could improve overall vaccination rates.

## 5. Conclusions

This study identified key determinants of COVID-19 vaccination among Korean adults using the Andersen model. Gender, age, education level, job status, marital status, psychological concerns about COVID-19, healthcare access, unmet healthcare needs, mental health conditions, and health behaviors all significantly shaped vaccination patterns. The findings suggest that public health authorities should focus on disseminating reliable information about vaccines, especially to economically disadvantaged individuals and those with poor mental or physical health. Tailored intervention strategies are necessary to actively promote vaccination uptake, particularly among socially vulnerable groups. In future research, longitudinal studies will be essential to monitor changes in vaccine perceptions and behaviors as the pandemic and vaccination campaigns evolve. Overall, this study contributes to the growing body of knowledge on COVID-19 vaccination and provides a solid foundation for designing educational and policy interventions to increase vaccination rates.

## Figures and Tables

**Table 1:** General characteristics of COVID-19 vaccination.

Variable		Total %	Unvaccinated %	Vaccinated %	*p*
Predisposing factors					
Age	19–64	65.7	71.4	65.5	<0.001
	≥65	34.3	28.6	34.5	
Gender	Male	45.8	43.9	45.8	<0.001
	Female	54.2	56.1	54.2	
Job status	Inoccupation	37.2	55.5	36.5	<0.001
	Occupation	62.8	44.5	63.5	
Spouse	Absence	34.1	42.9	33.8	<0.001
	Existence	65.9	57.1	66.2	
Education	≤High school	61.1	55.4	61.3	<0.001
	>College	38.9	44.6	38.7	
Psychological concerns of COVID-19					
Infection	Yes	64.5	60.3	64.7	<0.001
	No	35.3	39.7	35.3	
Public criticism	Yes	49.5	44.5	49.7	<0.001
	No	50.5	55.5	50.3	
Economic damage	Yes	54.1	50.6	54.2	<0.001
	No	45.9	49.4	45.8	
Enabling factors					
Unmet healthcare need	Yes	5.8	9.6	5.7	<0.001
	No	94.2	90.4	94.3	
Recipient of national basic livelihood guarantees	Yes	4.5	9.5	4.3	<0.001
	No	95.5	90.5	85.7	
Region of residence	Rural	43.5	39.1	43.6	<0.001
	Urban	56.5	60.9	56.4	
Need factors					
Subjective health	Below normal	59.8	64.8	59.6	<0.001
	Above good	40.2	35.2	40.4	
Stress	Yes	73.8	74.8	73.7	0.037
	No	26.2	25.2	26.3	
Depression	Yes	16.8	24.8	16.5	<0.001
	No	83.2	75.2	83.5	
Chronic disease	Yes	35.3	26.7	35.6	<0.001
	No	64.7	73.3	64.4	
Current smoking	Yes	16.4	18.2	16.3	<0.001
	No	83.6	81.8	83.7	
Current alcohol drinking	Yes	47.0	35.1	47.4	<0.001
	No	53.0	64.9	52.6	
Eating breakfast	Yes	33.9	39.6	33.7	<0.001
	No	66.1	60.4	66.3	

**Table 2:** Influencing factors of COVID-19 vaccination.

Categories		Univariate OR (95% CI)	*p*	Multivariate OR (95% CI)	*p*
Predisposing factors					
Age	19–64	1.31 (1.25–1.38)	<0.001	1.63 (1.52–1.74)	<0.001
	≥65	Reference		Reference	
Gender	Male	1.08 (1.03–1.3)	0.001	1.17 (1.11–1.24)	<0.001
	Female	Reference		Reference	
Job status	Inoccupation	0.46 (0.44–0.48)	<0.001	2.34 (2.21–2.47)	<0.001
	Occupation	Reference		Reference	
Spouse	Absence	1.47 (1.40–1.54)	<0.001	1.18 (1.12–1.24)	<0.001
	Existence	Reference		Reference	
Education	≤High school	1.28 (1.22–1.34)	<0.001	0.76 (0.72–0.81)	<0.001
	>College	Reference		Reference	
Psychological concerns of COVID-19					
Infection	Yes	1.21 (1.15–1.26)	<0.001	0.87 (0.82–0.92)	<0.001
	No	Reference		Reference	
Public criticism	Yes	1.23 (1.18–1.29)	<0.001	0.91 (0.86–0.97)	0.005
	No	Reference		Reference	
Economic damage	Yes	1.16 (1.11–1.21)	<0.001	1.01 (0.95–1.01)	0.766
	No	Reference		Reference	
Enabling factors					
Unmet healthcare need	Yes	0.57 (0.52–0.57)	<0.001	1.54 (1.41–1.67)	<0.001
	No	Reference		Reference	
Recipient of national basic livelihood guarantees	Yes	0.43 (0.40–0.46)	<0.001	1.59 (1.45–1.73)	<0.001
	No	Reference		Reference	
Region of residence	Rural	1.21 (1.15–1.26)	<0.001	0.97 (0.92–1.02)	0.177
	Urban	Reference		Reference	
Need factors					
Subjective health	Below normal	0.80 (0.77–0.84)	<0.001	1.27 (1.20–1.30)	<0.001
	Above good	Reference		Reference	
Stress	Yes	0.95 (0.90–1.00)	0.037	0.97 (0.92–1.03)	0.357
	No	Reference		Reference	
Depression	Yes	0.60 (0.57–0.63)	<0.001	1.36 (1.28–1.44)	<0.001
	No	Reference		Reference	
Chronic disease	Yes	1.52 (1.44–1.60)	<0.001	0.68 (0.64–0.72)	<0.001
	No	Reference		Reference	
Current smoking	Yes	1.14 (1.08–1.21)	<0.001	1.21 (1.13–1.30)	<0.001
	No	Reference		Reference	
Current alcohol drinking	Yes	1.67 (1.59–1.75)	<0.001	0.55 (0.52–0.58)	<0.001
	No	Reference		Reference	
Eating breakfast	Yes	Reference		Reference	
	No	0.77 (0.74–0.81)	<0.001	1.10 (1.04–1.16)	<0.001

## Data Availability

The datasets generated or analyzed during the study are available from the corresponding author upon reasonable request.
